# Performance of new synthesized emulsifiers in ecofriendly metal cutting fluid formulations

**DOI:** 10.1038/s41598-024-54636-2

**Published:** 2024-03-04

**Authors:** E. A. Elsharaky, M. R. Mishrif, A. S. El-Tabei, Amira E. El-Tabey

**Affiliations:** Egyptian Petroleum Research Institute (EPRI), Nasr City, Cairo 11727 Egypt

**Keywords:** Gemini emulsifiers, Cutting fluid additives, Emulsion stability, Contact angle, Corrosion test, Tribological properties, Chemistry, Chemical synthesis

## Abstract

This study aims to prepare mono and gemini nonionic emulsifiers differing in HLB to utilize in formulated metal cutting fluids. Also, the cationic gemini surfactant (GCS) was prepared and applied as a corrosion inhibitor and biocide in the formulations. FT-IR and NMR confirmed the chemical structure of the prepared compounds. Different oil package formulations were prepared by adding different trial concentrations of the additives (emulsifier, corrosion inhibitor, coupling agent, and biocide) to the eco-friendly vegetable oil (castor oil). Standard procedures were performed to assess the stability of the formulated base oil packages. Six Formulas demonstrated the greatest oil stability. Oil in water emulsions with varying formulated oil ratios (5–15 wt%) were prepared. A standard test was carried out to evaluate their performance as emulsion stability. It’s been demonstrated that Formulas II and V produced stable emulsions. The wettability alteration of formulas II and V on different metal surfaces was evaluated. The droplet size of formulated castor oil in water was determined via DLS. Corrosion test and tribological properties were also performed. The findings of this study indicate that Formula V is a good choice as a renewable addition for enhancing a variety of performance characteristics of the water-based cutting fluid.

## Introduction

The process of transforming a bulk metal into a component or part is known as metalworking. High pressure, high temperatures, and significant friction and tool wear are all factors in metalworking operations^1^. Due to various rising manufacturing costs, today’s designers and manufacturers are shifting away from traditional cooling systems and towards new cooling systems.

In the machinery and manufacturing sectors, metalworking fluids (MWFs), also known as metal cutting fluids (MCFs), are frequently used for cooling, lubrication, surface cleaning, rust prevention, flushes out chips to minimize the negative impacts of heat generated during machining, enhance workpiece surface quality, and extend tool life^2–8^.

Generally speaking, MCFs are produced by blending oil in water with the proper emulsifiers. Water and emulsifiable oils (soluble oils) combine to form milky emulsions. One of the soluble oils’ most important characteristics is how quickly and easily they can form stable emulsions. Such a fluid has the distinct advantage of combining the cooling properties of water and the lubricating properties of oil. Generally, the components of soluble oils are quite complex because they serve as stabilizing and coupling agents for two or more emulsifiers, rust inhibitors, lubricity-enhancing additives, and antibacterial defenses^9,10^. Emulsifying agents reduce interfacial tension, allowing smaller oil droplets to form in water with the same energy input. To enhance the interaction between the lipophilic components of the emulsifier and the oil, a coupler was added. In order to control or reduce the growth of microorganisms like bacteria, algae, and fungi, biocides are incorporated into the formulations of metalworking fluids. Corrosion inhibitors may be present in metalworking fluids to create a protective layer on machine parts and work-piece surfaces^11^. For a formulation of stable emulsifiable oils that lasts for a long time, the components must work in harmony. Low biodegradability of mineral oil results in environmental pollution^1^. The main adverse effect is due to improper usage, which causes air pollution, soil contamination, surface and groundwater contamination, and, as a result, contaminated agricultural products and food. Scientists and tribologists are currently looking into different substitutes for petroleum-based MWFs to address these problems. Vegetable oil-based MWFs are one example of such alternatives. Due to their greater environmental friendliness, renewable nature, lack of toxicity, and ease of biodegradation, vegetable oils are typically preferred to petroleum-based oils^12,13^. A new generation of cutting fluids with high machining performance and favorable environmental compatibility may also be developed with the use of vegetable oils and nontoxic additives^14–16^.

It is widely acknowledged from empirical evidence that machining operations are facilitated by supplying specific fluids to the area of the tool to work-piece contact. In fact, cutting fluids are used in the majority of real-world machining scenarios to extend tool life by limiting tool wear, lowering cutting resistance, offering a fine surface finish, and enhancing machining precision. The lubricating action of cutting fluids is due to the reduced shear stress over the entire chip-tool contact area because of the formation of a boundary lubrication layer prevents metallic contact between the chip and the tool. Cutting fluids application caused a steeper interfacial shear stress gradient, which reduced friction at the rake face and reduced chip curl radius. Cutting fluids shortens the distance between the chip and the tool in order to reduce the frictional force on the rake face^5^.

The main objective of this research is to prepare two different types of surfactants: three nonionic monomeric emulsifies, three gemini nonionic emulsifiers, and one cationic surfactant used as a corrosion inhibitor and biocide in the cutting oil formulas. The role of the prepared emulsifiers was investigated to evaluate the stability of the cutting fluid formulations. Then, investigate how metal-cutting fluid additives interact synergistically to produce high emulsion stability, wettability, anti-corrosion properties, and low friction coefficient as well as compare the data with the commercial ones.

## Materials and methods

### Materials

P-Hydroxy benzene sulfonic acid, dodecanoyl chloride, phosphorus pentachloride, triethylamine, 1,2 dibromoethane, and polyethylene glycol, PEG (200, 400, and 600), were supplied from Sigma Aldrich. All other chemicals were of a technical grade and were used as they were given. The physicochemical characteristics of castor oil are present in a former research paper^7^. The aqueous phase used to prepare MCFs is fresh tap water.

### Preparation of monomeric and gemini nonionic emulsifiers

#### Preparation of 4-(dodecanoyloxy) benzene sulfonic acid

The synthesis of 4-(dodecanoyloxy) benzene sulfonic acid and the chemical structure confirmed by spectroscopic analysis was previously described^17,18^.

#### Preparation of 4-(chlorosulfonyl) phenyl dodecanoate

4-(dodecanoyloxy) benzene sulfonic acid (0.01 mol) was refluxed with phosphorus pentachloride (0.012 mol) in xylene for 4 h. The solvent was removed by distillation, and the resulting 4-(chlorosulfonyl) phenyl dodecanoate was dried under vacuum^19,20^.

### Preparation of polyoxyethylene dodecanoyloxy benzene sulfonates (monomeric nonionic emulsifiers)

In the presence of dioxane, as solvent, 0.01 mol of 4-(chlorosulfonyl) phenyl dodecanoate was mixed with PEG with different molecular weights (200, 400, and 600). To the reaction mixture, a few drops of pyridine were added. After 2 h of stirring, the reaction was distilled and then purified by washing with diethyl ether^17,18^. Then 3 nonionic surfactants were obtained, EA, EB, and EC for that with PEG 200, PEG 400, and PEG 600, respectively.

### Preparation of gemini nonionic emulsifiers

**Figure 1:**
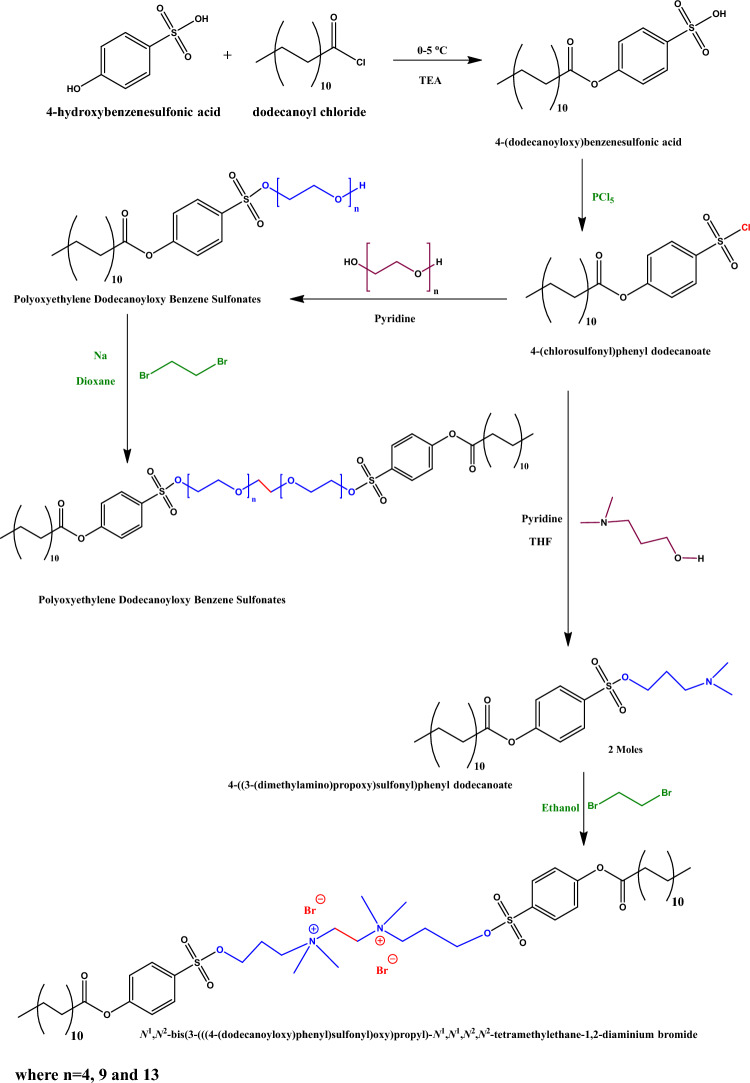
Preparation of the Monomeric, Gemini Nonionic Emulsifiers, and Cationic surfactant.

0.02 mol of Polyoxyethylene dodecanoyloxy benzenesulfonates (EA, EB, and EC) and 1,2 dibromoethane (0.01 mol) were combined in a round bottom flask with dioxane serving as the reaction’s solvent and sodium metal acting as the catalyst for 4 h^21–23^. Then vacuum distillation of the solvent was done, followed by diethyl ether washing to get the three nonionic gemini surfactants, GEA, GEB, and GEC. Figure 1 clearly illustrates the route for preparing nonionic gemini surfactants.

### Preparation of gemini cationic surfactant

#### Preparation of 4-((3-(dimethyl amino) propoxy) sulfonyl) phenyl dodecanoate

To a round bottom flask containing 3-(dimethylamino) propan-1-ol (0.01) and THF as the solvent, 0.01% 4-(chlorosulfonyl) phenyl dodecanoate was added drop by drop. Some drops of pyridine were added to the reaction mixture. The reaction was stirred for 2 h, after which the solvent was distilled and the product was washed with diethyl ether to prepare 4-((3-(dimethyl amino) propoxy) sulfonyl) phenyl dodecanoate^17,21^.

#### Preparation of N^1^,N^2^-bis(3-(((4-(dodecanoyloxy)phenyl)sulfonyl)oxy)propyl)-N^1^,N^1^,N^2^,N^2^-tetramethylethane-1,2-diaminium bromide

4-((3-(Dimethyl amino) propoxy) sulfonyl) phenyl dodecanoate was quaternized with 1,2 dibromoethane in the presence of ethanol for 24 h. The dissolved solvent was distilled off under a vacuum. The produced surfactant was re-crystallized from ethanol after being rinsed twice with diethyl ether to get the gemini cationic surfactant (GCS)^21–23^. The route for preparing the cationic gemini surfactant is shown in clear detail in Fig. 1.

### Antimicrobial activity of gemini cationic surfactant

The agar-well diffusion method was utilized to estimate the antimicrobial activity of the tested compound GCS^24–26^.

### Preparation of the cutting oil formulas

The cutting oil package was first created by combining the prepared emulsifiers or benchmark emulsifier (Tween based surfactant T-85), corrosion inhibitor, biocide, lubricant, and coupling agent with castor oil (vegetable base oil). A series of trial-and-error experiments were conducted to attain the optimum cutting oil package stability. cutting oil package in this study contains castor oil, a variety of prepared emulsifiers (EA, EB, EC, GEA, GEB, GEC, and T-85), a prepared corrosion inhibitor and biocide (GCS), lubricant oil (oleic acid), and coupling agent (dodecyl alcohol). Table 1 demonstrates how various proportions of these components were used to enhance the stability of cutting oil formulations.

**Table 1:** The Trial Range of Emulsifiable Cutting Oil Formulations.

Ingredients	Percentage of each component in cutting oil formula	
	Trial range, %	Optimum range, %
Castor oil	80–90	82
Emulsifiers (Monomeric, Gemini Nonionic and benchmark emulsifiers)	4–10	8
Lubricant oil (oleic acid)	1–5	4
Corrosion inhibitor and biocide (GCS)	1–3	3
Coupling agent (dodecyl alcohol)	2–5	3

### Assessment methods of cutting oil formulas

#### Hydrophile lipophile balance “HLB”

One of the factors that influence the choice of the best surfactants to use as emulsifying agents is the hydrophilic-lipophilic balance (HLB) within the molecule. The HLB value refers to the ratio of a surfactant molecule’s weight percentage of hydrophilic groups to that of lipophilic groups. On the HLB scale, which ranged from 0 to 20, the low end denoted an emulsifier that was significantly more soluble in oil than in water, while the high end denoted the opposite phenomenon. To put it another way, a low HLB value denotes a strong affinity for oil, whereas a high HLB value denotes a high water solubility. This means that while surfactants with HLB values of 3–8 stabilize W/O emulsion, those with HLB values of 9–12 stabilize O/W emulsion^27,28^.

The following formula can be used to determine the HLB^27,29^:

$$ {\text{HLB }} = {\text{ E}}/{5} $$

where E = stands for the percentage of ethylene oxide molecules in the molecules of the surfactant.

#### Stability of cutting oil formulations

In accordance with IP 311, the cutting oil package is put in a 100 ml covered glass test and stored at 50 °C as well as 0 °C for at least 15 h and no more than 20 h. The oil was immediately examined at the end of this period for any signs of turbidity, separation, or gelling. The blend that results in a homogeneous clear oil (with no gel formation or separation) was chosen for further study.

### Preparation of oil in water emulsions and evaluating their emulsion stability

In 10 ml graduated standard measuring tubes, the formulated cutting oils (5, 10, and 15%) and tap water (95, 90, and 85%) were mixed, respectively, to form the emulsions. At 25 °C, the test tubes were shaken vigorously for a period of 2 min. After 24 h, the resulting emulsion was visually checked for stability (oil and/or cream separations) via the standard test method (IP 263). Only the emulsion that demonstrated exceptional emulsion stability (ml oil/ml cream = 0/0) was chosen for additional testing. An emulsion was deemed to “Pass” if the total separation (oil and/or cream) after standing 24 h was less than 0.1 ml.

#### Surface and interfacial tension measurements

The surface tension of the prepared mono and gemini nonionic emulsifiers and various formulations was measured using the pendant drop technique utilizing Theta optical tensiometer, Biolin Scientific Company, Finland at 25 °C (298 K). Also, the interfacial tension for castor oil containing the prepared emulsifiers and water was measured.

#### Contact angle or wettability evaluation

Theta optical tensiometer, Biolin Scientific Company, Finland, was used to measure the contact angle between various metals (Aluminum [Al], Carbon Steel [CS], and Tungsten Carbide [WC]) and the various Formulas at 25 °C (298 K).

#### Droplet size of dispersed castor oil

At 25 °C, the oil droplet size distribution of the prepared emulsions formulas was determined utilizing dynamic light scattering (Malvern Zetasizer ZS, Worcestershire, UK).

#### Photographic studies of the prepared cutting oil emulsions

At 25 °C (298 K), a ZEISS Axiolab 5 digital laboratory optical polarizing microscope equipped with **a** Leica MC190 HD microscope camera was used to conduct photographic microscopy studies on the prepared emulsions’ formulas. An emulsion droplet was spread and exanimated on a glass slide.

#### Corrosion test on iron chips

This test was conducted using the ASTM D4627-92 standard test method, putting 5 ml of metal cutting emulsion on 4 gm of cleaned cast iron chips on filter paper in a covered glass container. Benzene was used to clean the cast iron chips prior to use. After 24 h, the anti-rust effect could be seen visually. The degree of rust on the filter paper was used to gauge how well the substance prevented rust. The corrosion level was determined via a 0–10 scale. No rust is marked with a 10, one spot of rust was marked with a 9, two spots with an 8, some spots with a 7, many spots with a 6, and numerous spots with rust and stains were marked with a 5^30–32^.

#### Tribology test

A tribology measuring cell connected to Physica MCR-502 controlled-stress rheometers was used to conduct tribological tests^33–35^. A ball-on-pyramid principle governs the setting of the tribology accessory^36^. A 6.35 mm diameter steel ball (1.4401 grade 100) rotating on three steel plates (1.4301) at a 45° angle is used in the cell. The evolution of the friction coefficient with the sliding distance was tested for normal load 10 and 900 s at 25 °C.

## Result and discussion

### Chemical structure’s elucidation

The prepared compounds’ structures were verified using FT-IR and NMR spectra. The first step for preparing 4-(dodecanoyloxy) benzene sulfonic acid was elucidated in a former research paper^18^.

#### Confirmation of the chemical structure of the prepared 4-(chlorosulfonyl) phenyl dodecanoate

**Figure 2:**
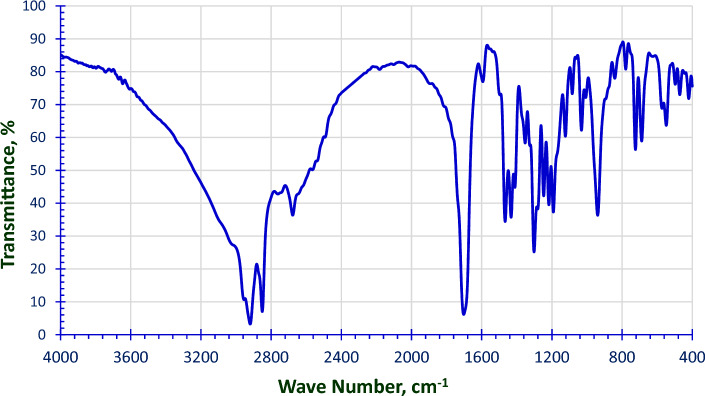
FT-IR Spectra for the Prepared 4-(Chlorosulfonyl) Phenyl Dodecanoate.

Figure 2, shows FTIR peaks (ν_max_, cm^−1^) at 2919.3 & 2850.5 cm^−1^ for (**CH**_**3**_ & **CH**_**2**_ stretch), 1702.7 cm^−1^ (**C=O**), 1593.7 cm^−1^ **(Ar C–C)**, 1432.6 cm^−1^ **(Ar C=C)**, 1352.6 cm^−1^ (**Cl–S=O** asym. str.) and 1191.8 cm^−1^ (**Cl**–**S=O** sym. str.).

#### Confirmation of the chemical structure of the prepared monomeric and gemini nonionic emulsifiers

- The chemical structure of polyoxyethylene 600 dodecanoyloxy benzene sulfonate (EC) was confirmed using FT-IR and ^1^HNMR.

**Figure 3:**
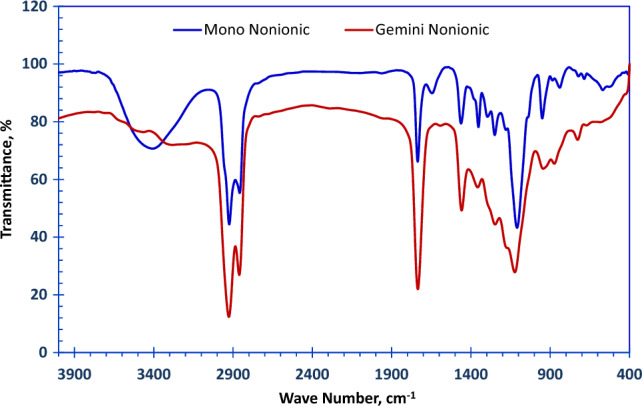
FT-IR Spectra for the Prepared (**a**) Monomeric Nonionic Emulsifier (EC) and (**b**) Gemini Nonionic emulsifier (GEC).

Figure 3a, displays the appearance of some new characteristic bands in the FTIR spectra, **OH** stretch (broad band) at 3407.02 cm^−1^, ethereal band **C–O–C** asym. stretch at 1109.0 cm^−1^, **S=O** asym. stretch at 1352.5 cm^−1^ and **S=O** sym. stretch at 1249.7 cm^−1^.

**Figure 4:**
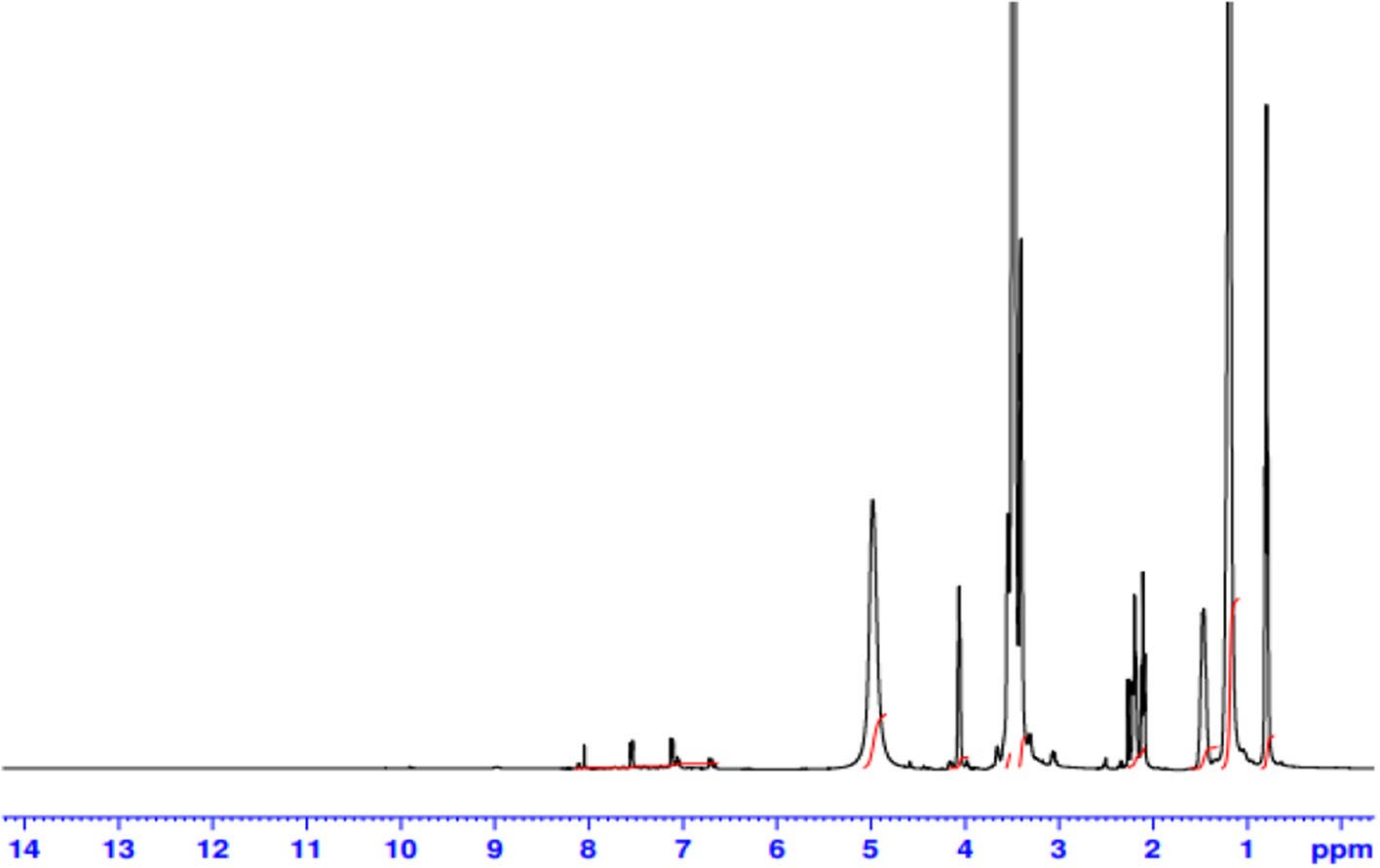
^1^H NMR Spectra for the Prepared Monomeric Nonionic Emulsifier (EC).

Figure 4 demonstrates the ^1^H-NMR of [EC]. Peaks assignment can be collected as follows: (a) δ = 0.80 due to the proton of –C**H**_3_; (b) δ = 1.18 for –(C**H**_2_)_8_– ; (c) δ = 1.49 for C**H**_2_CH_2_COOR; (d) δ = 2.20 for CH_2_C**H**_2_COOR; (e) = 3.52 for –OC**H**_2_CH_2_OC**H**_2_CH_2_OH; (f) δ = 3.70 for –OCH_2_CH_2_OCH_2_C**H**_2_OH; (g) δ = 4.06 for –SO_3_C**H**_2_CH_2_–; (h) δ = 4.98 for –OCH_2_CH_2_OCH_2_CH_2_O**H**; (h) δ = 7.10—8.04 for different protons of the benzene ring.

b) The chemical structure of Gemini polyoxyethylene 600 dodecanoyloxy benzene sulfonate (GEC) was confirmed by FT-IR and 13C NMR.

The FT-IR of Gemini nonionic surfactant (GEC) as a representative sample is obvious in Fig. 3b. It clears the disappearance of the broad band of hydroxyl group of the PEG which confirms the formation of the Gemini surfactant.

**Figure 5:**
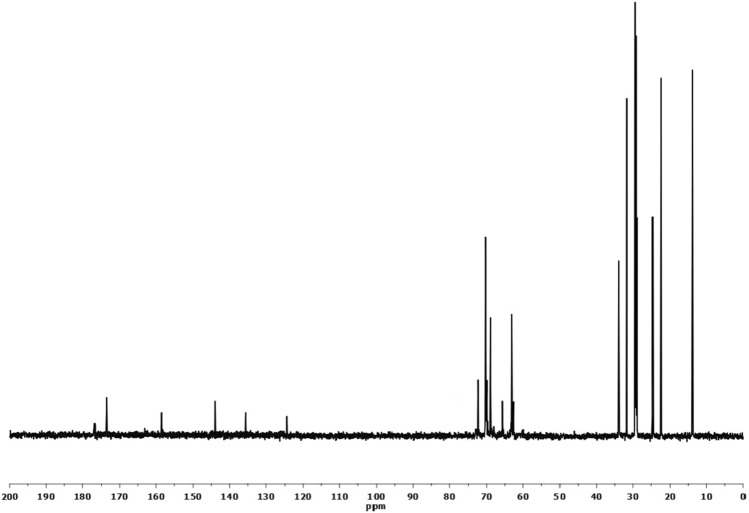
^13^C NMR Spectra for the Prepared Gemini Nonionic Surfactant (GEC).

The peak assignment of ^13^C-NMR (Fig. 5) can be collected as follows: (a) δ at 13.8 ppm for –CH_2_–**C**H_3_ protons, (b) δ at 22.4 ppm for CH_3_–**C**H_2_(CH_2_)_n_, (c) δ at 31.7 ppm for CH_3_–CH_2_ **C**H_2_(CH_2_)_n_, (d) δ at 29.4 ppm for CH_3_–CH_2_(**C**H_2_)_n_, (e) δ at 24.6 ppm for –**C**H_2_–CH_2_–COO–, (f) δ at 33.7 ppm for –CH_2_–**C**H_2_–COO–, (g) δ at 173.5 ppm for –CH_2_–**C**OO–, (h) δ at 158.3 ppm for –COO–**C**=CH–, (i) δ at 124.5 ppm for –COO–C=**C**H–, (j) δ at 135.4 ppm for –O_3_S–C=**C**H–, (k) δ at 143.9 ppm for –O_3_S–**C**=CH–, (l) δ at 63.1 ppm for –O_2_SO–**C**H_2_CH_2_O–, (m) δ at 65.8 ppm for –O_2_SO–CH_2_**C**H_2_O (n) δ at 70.3 ppm for –O_2_SO–(**C**H_2_**C**H_2_O)_n_–.

#### Confirmation of the chemical structure of the prepared gemini cationic surfactant

- The chemical structure of 4-((3-(dimethyl amino) propoxy) sulfonyl) phenyl dodecanoate was elucidated utilizing FT-IR and ^1^HNMR.

**Figure 6:**
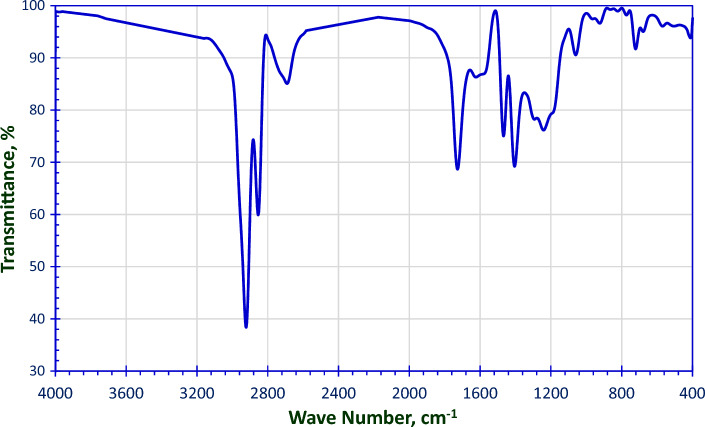
FT-IR Spectra for 4-((3-(Dimethyl Amino) Propoxy) Sulfonyl) Phenyl Dodecanoate.

Figure 6 depicts the FT-IR of 4-((3-(dimethyl amino) propoxy) sulfonyl) phenyl dodecanoate. The characteristic bands are **S=O** asym. stretch at 1405.1 cm^−1^ and **S=O** sym. stretch at 1241.3 cm^−1^ indicating the presence of the sulphonate group “–**SO**_**3**_–”.

**Figure 7:**
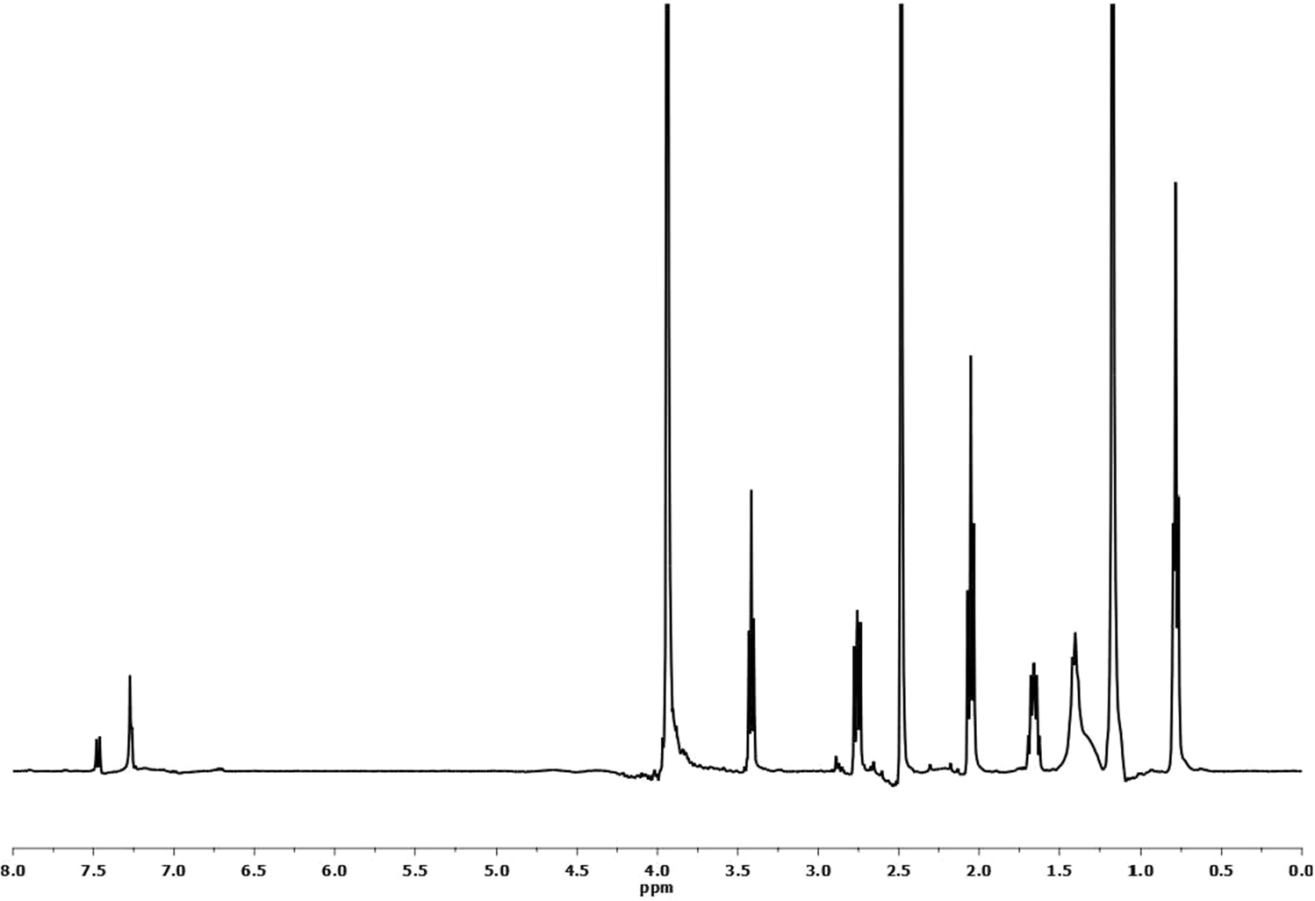
^1^H NMR Spectra for 4-((3-(Dimethyl Amino) Propoxy) Sulfonyl) Phenyl Dodecanoate.

^1^H NMR of the prepared 4-((3-(dimethyl amino) propoxy) sulfonyl) phenyl dodecanoate was performed to confirm its structure as shown in Fig. 7. From this figure: peaks can be gathered as follows: (a) δ at 0.78 ppm for –CH_2_–C**H**_3_ protons, (b) δ at 1.16 ppm for (C**H**_**2**_)_8_, (c) δ at 1.40 ppm for –C**H**_**2**_–CH_2_–COO, (d) δ at 2.73 ppm for –CH_2_–C**H**_**2**_–COO, (e) δ at 2.05 ppm for –CH_2_–CH_2_–C**H**_2_–N–(CH_3_)_2_, (f) δ at 1.65 ppm for –CH_2_–C**H**_**2**_–CH_2_–N–(CH_3_)_2_, (g) δ at 3.4 ppm for –C**H**_**2**_–CH_2_–CH_2_–N–(CH_3_)_2_, (h) δ at 2.48 ppm for –CH_2_–CH_2_–N–(C**H**_3_)_2_ and (i) δ at 7.26–7.48 for various benzene ring protons.

**Figure 8:**
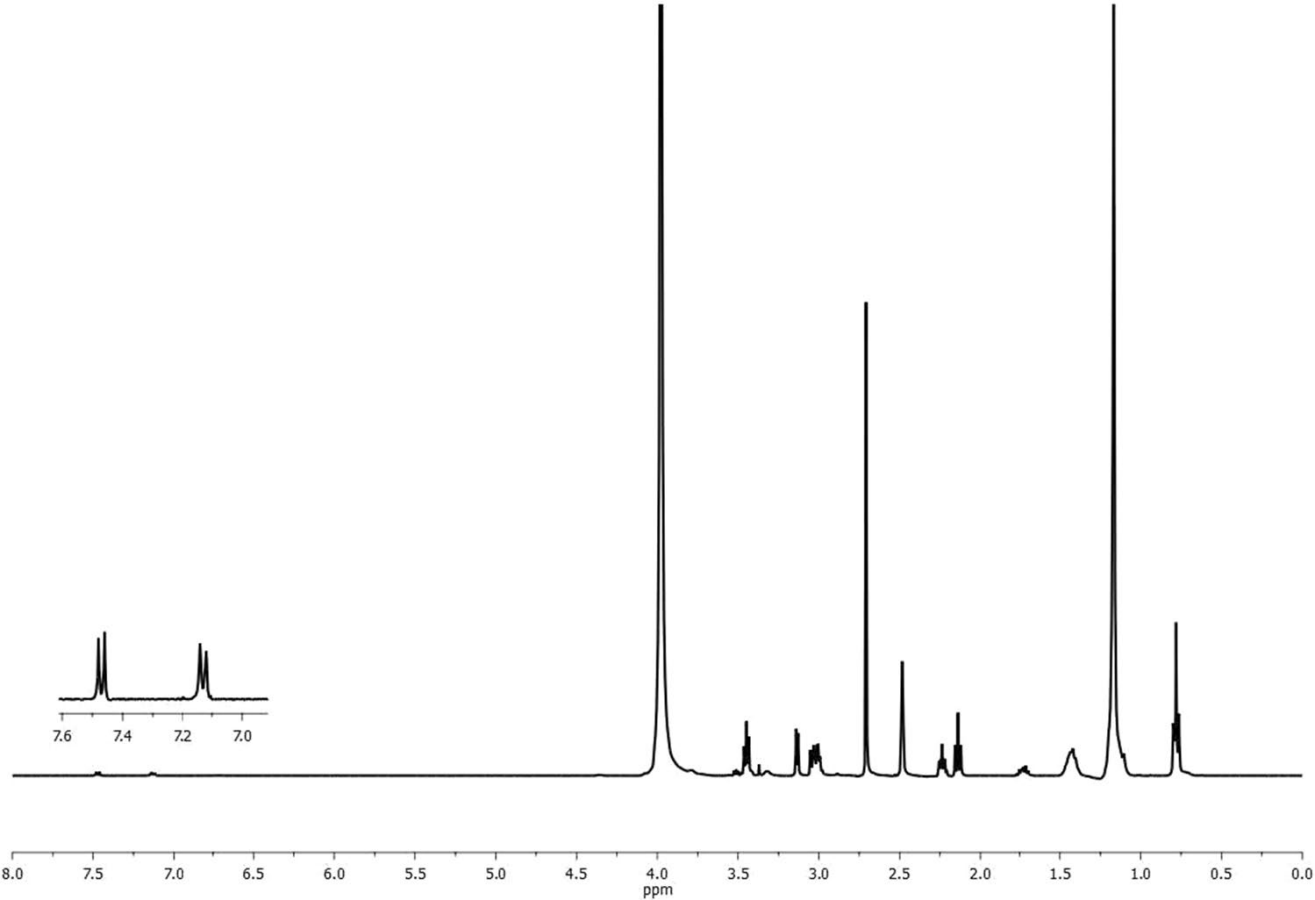
^1^H NMR Spectra for the Prepared Gemini Cationic Surfactant (GCS).

b) The chemical structure of Gemini cationic surfactant (GCS) was confirmed using ^1^H NMR. The ^1^H NMR’s peaks assignments in Fig. 8 for GCS can be gathered as follows:

δ at 0.78 ppm for –CH_2_–C**H**_3_ protons, (b) δ at 1.17 ppm for (C**H**_**2**_)_n_, (c) δ at 1.4 ppm for –C**H**_**2**_–CH_2_–CH_2_–COO, (d) δ at 1.7 ppm for –C**H**_**2**_–CH_2_–COO, (e) δ at 2.1 ppm for –O–CH_2_–C**H**_**2**_–CH_2_–N–(CH_3_)_2_, (f) δ at 2.23 ppm for –C**H**_**2**_–COO, (g) δ at 2.7 ppm for –CH_2_–CH_2_–N^+^–(C**H**_**3**_)_2_, (h) δ at 3.0 ppm for –CH_2_–C**H**_**2**_–N^+^–, (i) δ at 3.14 ppm for the spacer –CH_2_–CH_2_–N^+^–C**H**_**2**_–C**H**_**2**_–N^+^–, (j) δ = 3.45 for –SO_3_C**H**_2_CH_2_–, and (k) δ at 7.1–7.4 for phenyl ring protons.

### Surface active properties of the prepared surfactants

#### Surface tension (γ)

**Figure 9:**
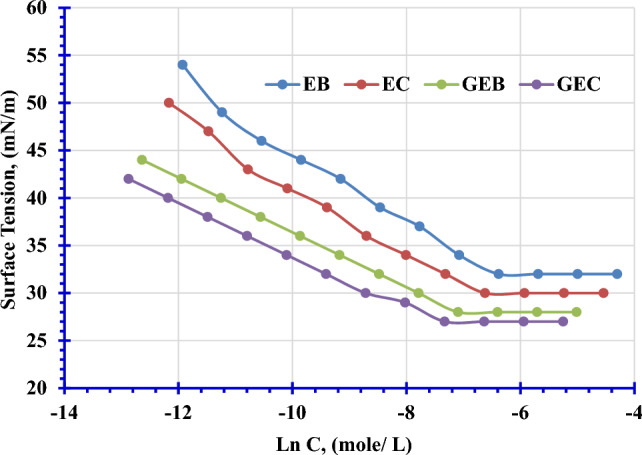
Variation of the Surface Tension with Concentrations of Prepared Surfactants in Distilled Water at 25 °C.

Figure 9 exhibits the curve of surface tension (γ) versus the log concentration for the prepared mono- and gemini nonionic surfactants. These curves demonstrate that even at low concentrations of surfactants, their molecules can be adsorbed at the interface, causing a decrease in the surface tension of water^37^. Also, Fig. 9 illustrates a steep linear decline in surface tension as the concentration of surfactants increases up to the critical micelle concentration (CMC). Beyond the (CMC), surface tension remains constant even with further increases in surfactant concentration. This indicates that, beyond the CMC, additional surfactant concentration does not affect surface tension, as depicted in the Fig. 9. The observed reduction in surface tension is attributed to the formation of a monolayer by the surfactant adsorbed at the air/liquid interface. Consequently, there are no more spaces for adsorption at the interface beyond the CMC, and the surfactant molecules begin to aggregate into micelles^38^. The surface tension values for the prepared surfactants, EB, EC, GEB, and GEC decrease in the order of 32 > 30 > 28 > 27 mN/m respectively. Gemini surfactants exhibited a higher decrease in surface tension values than the corresponding monomeric surfactants; this might be attributed to increased hydrophobicity content.

#### Critical micelle concentration (CMC)

Table 2 lists the critical micelle concentration (CMC) values determined from Fig. 9. The CMC values for the prepared mono- and gemini nonionic surfactants, EB, EC, GEB, and GEC decrease in the order 0.00169 > 0.00133 > 0.000829 > 0.000655 mol/L, respectively. This might be due to the oxyethylene content increased. Gemini surfactants exhibit lower CMC values than monomeric surfactants, which might be attributed to the duplication of oxyethylene units and hydrophobicity.

**Table 2:** Surface Active Properties of the Prepared Monomeric and Gemini Nonionic Surfactants at 25 °C.

Surfactant	γ (mN m^−1^)	CMC (mol dm^−3^)	*π*_cmc_ (mN m^−1^)	*Γ*_max_ × 10^10^ (mol cm^−2^)	A_min_ (A°^2^ molecule^−1^)	∆ G_mic_ (kJ mol^−1^)	∆G_ads_ (kJ mol^−1^)
EB	32	0.00169	40.3	1.55	107.35	− 15.81	− 18.41
EC	30	0.00133	42.3	1.48	112.03	− 16.40	− 19.25
GEB	28	0.000829	44.3	1.16	142.76	− 17.57	− 21.38
GEC	27	0.000655	45.3	1.11	148.97	− 18.15	− 22.22

#### Maximum surface pressure (π_cmc_)

Based on the information provided in Table 2, it appears that the maximum surface pressure (π_cmc_) is a measure of the surface activity of a surfactant at the critical micelle concentration (γ_cmc_) compared to distilled water (γ_0_). Lower values of π_cmc_ indicate lower surface activity, while higher values suggest greater surface activity^39^.

The table provided shows the surface pressure values for various monomeric and gemini nonionic surfactants. In general, the monomeric nonionic surfactants have lower surface pressure values than the gemini nonionic surfactants, indicating that they are less surface-active. Within each series (monomeric and gemini nonionic) of surfactant, The surface pressure values increase with values 40.3, 42.3, 44.3, and 45.3 for EB, EC, GEB, and GEC, respectively.

The statement that increasing surface pressure values indicate a higher collection of surfactant molecules at the interface suggests that surfactant molecules are adsorbing onto the surface and forming a monolayer. The higher the surface pressure, the more tightly packed the surfactant molecules are at the interface^39^.

#### Surface excess concentration (Γ_max_)

The surface excess concentration (Γ_max_) of surfactants at surface saturation is an important parameter that indicates the effectiveness of a surfactant’s adsorption at interfaces^40,41^. Table 2 presents data on Γ_max_ for the prepared monomeric and gemini nonionic surfactants. For monomeric and gemini nonionic surfactants, the order of increasing Γ_max_ is 1.55 × 10^–10^ < 1.48 × 10^–10^ < 1.16 × 10^–10^ < 1.11 × 10^–10^ mol/cm^2^ for EB, EC, GEB, and GEC, respectively. The data also reveal that gemini surfactants have lower Γ_max_ values than comparable monomeric surfactants.

#### Minimum area per molecule (A_min_)

A_min_ provides information about the packing and orientation of the adsorbed surfactant molecules. The data in Table 2 demonstrates that lower Γ_max_ values lead to parallel coverage at the interface, resulting in higher A_min_ values^42^.

For the prepared monomeric and gemini nonionic surfactants, the order of increasing A_min_ values is as follows: 107.35 < 112.03 < 142.76 < 148.97 A^°2^/molecule for EB, EC, GEB, and GEC, respectively.

#### Free energy of micellization and adsorption

Table 2 reveals that both micellization and adsorption processes have negative free energy values (∆G_mic_ and ∆G_ads_), indicating that they are spontaneous. However, the ∆G_ads_ value is higher in negativity than the ∆G_mic_ value, indicating that molecules tend to preferentially adsorb onto the interface. The prepared monomeric and gemini nonionic surfactants exhibit the following order of ∆G_mic_ values: (− 15.81 > − 16.40 > − 17.57 > 18.15 kJ/mol) for EB, EC, GEB, and GEC, respectively. The order of ∆G_ads_ values is (− 18.41 > − 19.25 > − 21.38 > − 22.22 kJ/mol) for EB, EC, GEB, and GEC, respectively.

#### Interfacial tension (IFT) measurements

**Figure 10:**
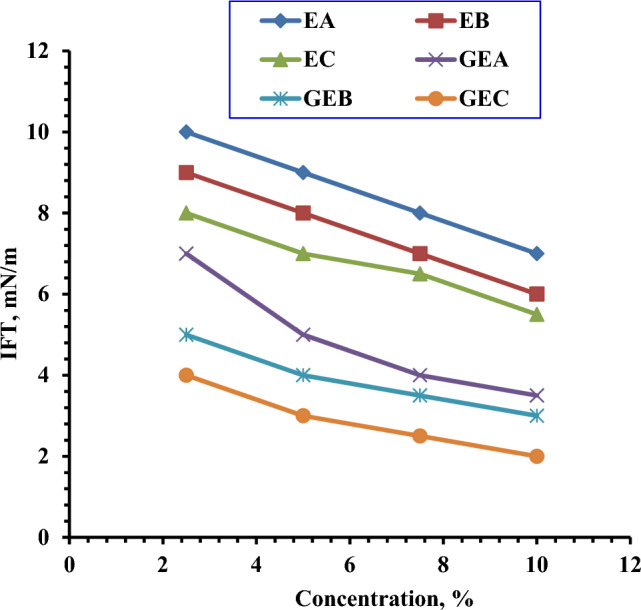
Variation of the Interfacial Tension with Concentrations of Prepared Surfactants at 25 °C.

IFT was determined for monomeric and gemini nonionic surfactants at concentrations of 2.5, 5, 7.5, and 10% as shown in Fig. 10. According to the results, the IFT values of gemini surfactants are lower than the IFT values of monomeric surfactants. This might be attributed to an increase in the number of oxyethylene units and the length of the alkyl chain length. The IFT values decline as concentrations increase (Figure 10).

**Table 3:** The biocidal activity of the GCS toward Gram—positive bacteria, Gram—negative bacteria, Yeast and Fungi.

Sample	Gram—positive bacteria		Gram—negative bacteria		Yeast and Fungi	
	*Bacillus subtilis*	*Staphylococcus aureus*	*Escherichia coli*	*Pseudomonas putida*	*Candida albicans*	*Aspergillus niger*
GCS	21	20	20	18	17	17
Reference	31	33	23	29	27	26

### Antimicrobial activity of gemini cationic surfactant

Water is added to the cutting fluid formulations to aid in the cooling and lubrication of the cutting tools during machining operations. However, water can also serve as a source of microbial contamination, as it provides a suitable environment for the growth and proliferation of microorganisms. Microbial contamination of soluble metal cutting fluids can lead to a range of issues, including reduced tool life, poor surface finish, and adverse health effects for workers exposed to the contaminated fluids. Therefore, it is essential to monitor and control the microbial contamination of these fluids to ensure their safety and efficacy. One approach to controlling microbial contamination in soluble metal cutting fluids is through the use of biocides. Biocides are chemical agents that are added to the cutting fluid to inhibit the growth and proliferation of microorganisms^43^. Table 3 displays that GCS has a notable ability to inhibit all tested microbial strains, with inhibition zones (mm) ranging from 18 to 21 mm for all types of bacteria and 17 mm for fungi and yeast species. These values demonstrate a significant antimicrobial effect of the gemini compared to control values. The mechanism of action of cationic surfactants involves their positively charged head groups interacting with the negatively charged components of the cell membrane, such as the phosphate groups of phospholipids and lipopolysaccharides. The interaction prompts the surfactant molecules to accumulate at the surface of the cell, where they can penetrate the membrane and cause its disruption, resulting in cell death^22^.

**Table 4:** Oil package stability, emulsion stability and surface tension test of different metal Cutting Fluids Formulas.

Test	Formula I (EA)	Formula II (EB)	Formula III (EC)	Formula IV (GEA)	Formula V (GEB)	Formula VI (GEC)	Formula VII (T-85)
Oil stability (IP 311)	Clear and homogenous						
Emulsion stability, (IP 263)							
Oil / Cream, mL							
a) O/W = 5/95	−Ve*	Nil/Nil	0/1	1/0	Nil/Nil	0/0.5	Nil/Nil
b) O/W = 10/90	−Ve	Nil/Nil	0/2	2/0	Nil/Nil	0/1	Nil/Nil
c) O/W = 15/85	−Ve	Nil/Nil	0/2	2/0	Nil/Nil	0/1	Nil/Nil
Surface tension (γ) (5 O/95 W)	–	33.41	–	–	29.93	–	31.56

### Assessment methods of cutting oil formulas

#### The stability of the formulated oil package

**Figure 11:**
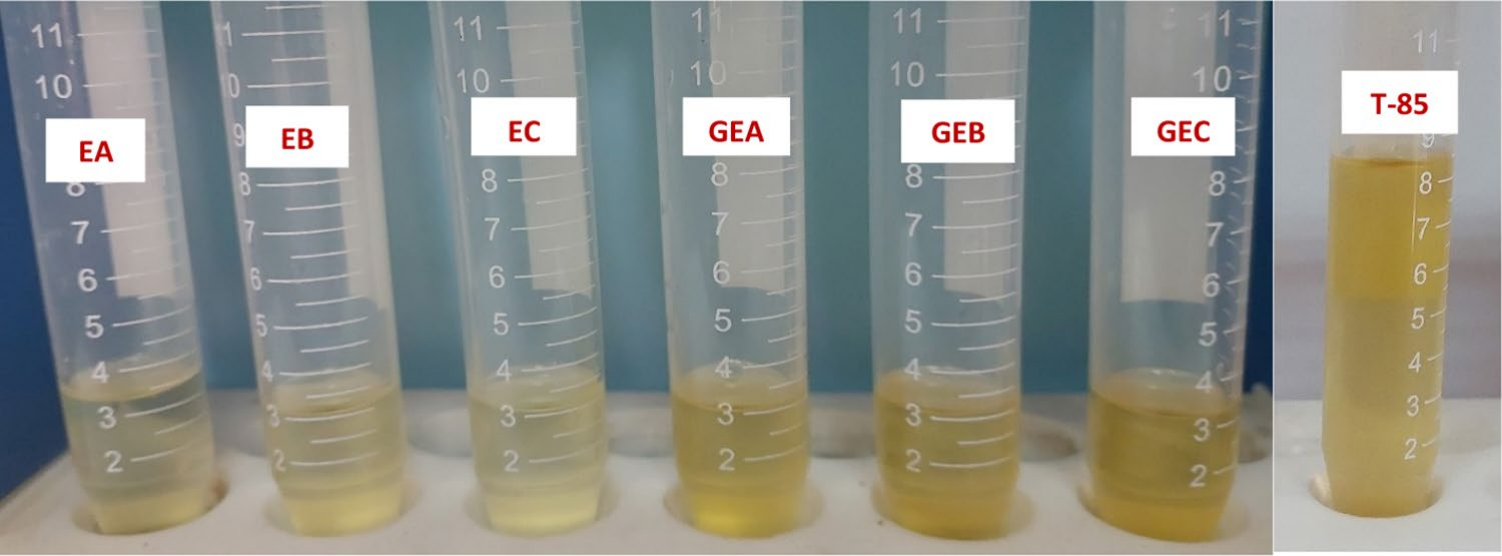
Oil Stability for the Cutting Oil Package Containing Nonionic Emulsifiers (EA, EB and EC), Gemini Nonionic Emulsifiers (GEA, GEB and GEC) and Benchmark Emulsifier (T-85).

A number of cutting oil formulas with varying concentrations of castor oil (non-edible vegetable oil), emulsifiers (prepared and benchmark emulsifiers), corrosion inhibitor, biocide, lubricant oil, and coupler were formulated as shown in Table 4. These formulations were created in order to investigate the effect of cutting oil ingredients on the stability of both the cutting oil package and the MCF emulsion. At 0 and 50 °C, the stability of the oil formulations was examined. The formula that produces a stable cutting oil package (free of gel formation, turbidity evidence, or separation) was chosen for additional investigations, as shown in Fig. 11. According to the preliminary findings shown in Table 4, in order to form stable cutting oil, the optimum percentages by volume of cutting oil ingredients are 82% castor oil, 8% emulsifier(prepared and benchmark emulsifiers), 3% corrosion inhibitor and biocide, 3% coupling agent and 4% lubricant oil (oiliness).

#### Emulsion stability of cutting oil formulas

For this purpose, eighteen cutting fluids were prepared by combining different water contents of 85, 90, and 95 wt% with various cutting oil concentrations of 15, 10, and 5 wt%, respectively, at 25 °C for 24 h. Seven emulsifiers **EA** (in **Formula I**), **EB** (in **Formula II**), **EC** (in **Formula III**), **GEA** (in **Formula IV**), **GEB** (in **Formula V**), **GEC** (in **Formula VI**), and **T-85** (in **Formula VII**) were used to prepare these cutting fluid formulas.

**Figure 12:**
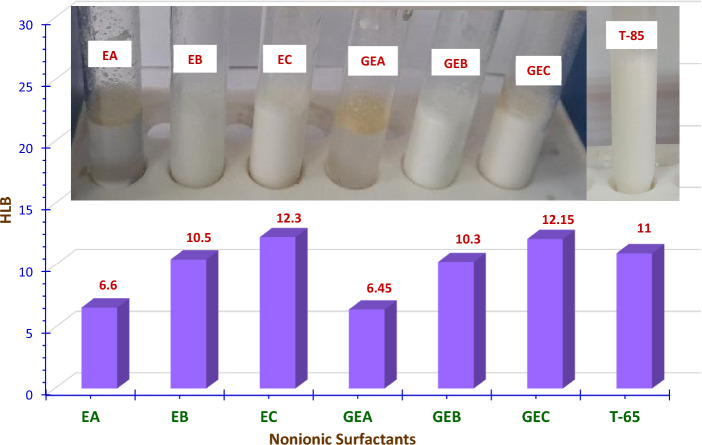
HLB values for the Prepared Monomeric, Gemini Nonionic Emulsifiers, Benchmark Emulsifier (T-65) and Emulsion Stability for the Cutting Oil Package Containing the Prepared Nonionic Emulsifiers and T-65.

The results presented in Table 2 and Fig. 12 demonstrate that **Formula II**, **Formula V, and Formula VII** exhibit good emulsion stability at the three concentrations (Nil/Nil (stable emulsion formed)). Whereas, **Formula III** and **Formula VI** give creaming layer separation. The thickness of this layer increases with an increase in oil package concentration. **Formula I**, on the other hand, is unable completely to produce an emulsion (− Ve (no emulsion formed)), whereas, **Formula IV** separates an oil layer, and this layer increases as oil content increases as lucid in Table 2 and Fig. 12.

### Effect of hydrophile lipophile balance “HLB” of the prepared emulsifiers on emulsion stability

The kind of prepared emulsion is greatly influenced by the surfactant’s HLB value, making it a crucial variable in the emulsion preparation process. Accordingly, choosing an appropriate HLB value is necessary to create an emulsion with a particular set of properties^27–29^. Three mechanisms: creaming, flocculation, and coalescence, are generally thought to control the emulsion separation process^7^.

As can be seen in Fig. 12 and Table 2, for the three percentages of (oil package: water), without creaming layer separation, the best stability of O/W emulsions was obtained at HLB value of 10.5 for **EB,** 10.3 for **GEB,** and 11.0 for **T-85**, in **Formulas II, V**, and **VII** respectively. This could be attributed to the efficient partitioning of surfactant molecules between the water and oil, which helps to form a rigid interface film around the castor oil droplet. In turn, this results in a reduction of the interfacial tension between oil and water. Thus, the likelihood of castor oil droplets flocculating and coalescing was reduced^7,44^. Formula **III** and **VI,** on the other hand, were excluded because of the presence of a very thin creamy layer on the top. This may be because of the HLB value for **EC** (HLB = 12.3) and **GEC** (HLB = 12.15) used in **Formula III** and **VI** respectively. This might be because the minimal coalescence is occurring at a typical HLB value, specifically 12.0 for O/W emulsion in the investigated emulsions^44,45^. Also, **Formulas I** and **IV** with **EA** and **GEA** surfactants, respectively, will be excluded because they are unable to create an emulsion (HLB = 6.6 and 6.45 respectively). Since a low HLB number suggests a strong oil affinity. This means that surfactants with HLB values of 3–8 stabilize W/O emulsion and not O/W emulsion^28^.

From the emulsion stability test, only the soluble oil blends that demonstrated exceptional emulsion stability in terms of having no separated oil and no cream after 24 h were chosen for the subsequent tests. Hence, **Formula II, Formula V,** and **Formula VII** were then selected to undergo further tests. From an economic standpoint, the ratio of 95% water to 5% cutting oil was chosen to conduct the further tests because **Formula II**, **Formula V,** and **Formula VII** display outstanding emulsion stability at the three concentrations of water to cutting oil content (95, 90, and 85 wt%: 5, 10, and 15 wt%).

#### Surface tension of formulated metal cutting fluids

Emulsifying agents work to reduce surface tension, which makes it possible to generate smaller oil droplets in water using the same amount of energy. Additionally, emulsifiers inhibit or delay droplet flocculation and coalescence by creating an interfacial film around the dispersed phase oil droplets^46^. The boiling process, wetting behavior, and spray characteristics of fluid are all significantly influenced by surface tension^47^. **Formula V** (Surface Tension = 29.93 mN/m) has a lower surface tension value than **Formula II** and** Formula VII** (Surface Tension = 33.41 and 31.56 mN/m), as lucid in Table 4, which might be due to the solubilization effect.

#### Wettability of formulated metal cutting fluids

**Figure 13:**
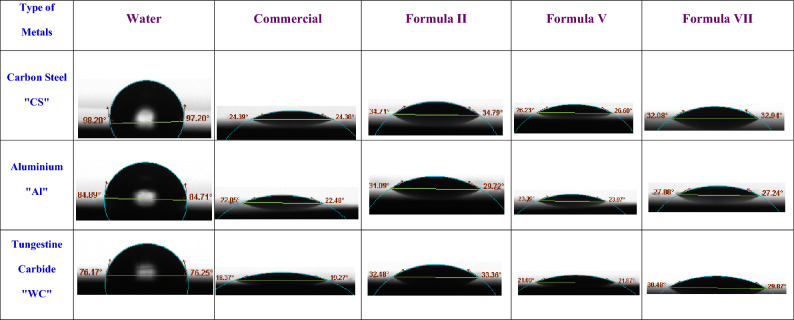
Contact Angle of water, commercial sample, Formula II, Formula V and Formula VII on Different Metals: Carbon Steel (CS) and Aluminium (Al) as the Work Piece whereas, Tungestin Carbide (WC) as the cutting tool.

Lubrication of the tool’s and workpiece’s surface is primarily influenced by the wettability of MCFs^48^. Wettability is the term used to describe a liquid’s capacity to cover, encircle, and penetrate the cutting tool and workpiece. It also relates to how well a fluid performs when it comes to machining. The wetting ability of a liquid is an indication of its attraction to the solid surfaces^49^. The contact angle between a liquid droplet and a solid surface is what determines a fluid’s wettability. Typically, the surface tension of the fluid has a direct relationship with the wettability^47,50^. To simulate the carbon steel (CS) and aluminum (Al)-based workpiece, and also the tungsten carbide (WC) cutting tool material, the contact angle was measured for the emulsion formulations on these surfaces. Between tests, the sample surfaces were cleaned with ethanol and then dried. Figure 13 shows the wetting angles for water, commercial sample, **Formula II, Formula V,** and** Formula VII** cutting fluids formulas on the workpieces (Al and CS), and tool (WC). The contact angle values on CS, Al, and WC are 97.20, 84.71, and 76.25 °C for water; 24.38, 22.48 and 19.27 °C for the commercial sample; 34.79, 29.72 and 33.36 °C for **Formula II**; 26.60, 23.97 and 21.87 °C for **Formula V**, and 32.94, 27.24 and 29.87 °C for **Formula VII** respectively. According to Fig. 13, **Formula V** has higher wettability because it can wet the surface more thoroughly. This implies that **Formula V** has the best wettability on the workpiece and tool.

#### Droplet size and photographic studies of formulated cutting fluids

**Figure 14:**
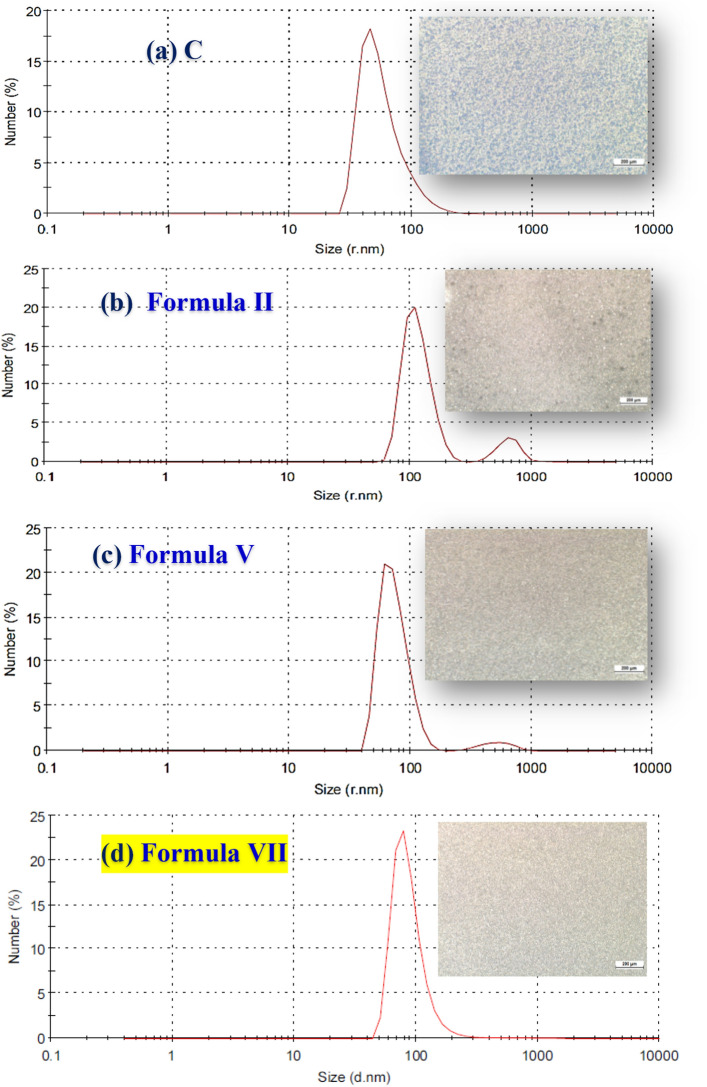
Dynamic Light Scattering (DLS) and Photographic Microscopic Date for Oil in Water Emulsion utilizing (**a**) Commercial Sample, (**b**) Formula II, (**c**) Formula V and (**d**) Formula VII.

Figure 14 displays the emulsion’s droplet size for **a commercial sample**, **Formula II**, **Formula V,** and **Formula VII**. It is obvious that an oil–water emulsion forms with an oil droplet size of 116.2 nm, 74.3, and 91.95 nm when using **Formula II**, **Formula V,** and **Formula VII** respectively. It is worth noting that the droplet size of **Formula V** is smaller than that of **Formula II** and **Formula VII**. **Formula V** has a high propensity for adsorption because of its gemini emulsifier structure, which reduces vegetable oil droplet size and increases the intensity of the adsorptive film surrounding the oil droplet. As a result, a greater intensity of the surfactant film prevents oil droplet aggregation. Additionally, it can be seen that the droplet size of the **Formula V** is close to that of the commercial sample (58.7 nm). The dispersed oil droplets in the photographic images in Fig. 14 appear to be evenly distributed throughout the continuous water phase. It is important to note that the nano-size of the droplets is a factor in increasing the emulsion stability. This may be because of the consistency of the droplets which prevents creaming. Since, differences in droplet sizes may promote the Oswald ripening mechanism, which causes the droplets to ripen and then separate forming a creamy or oily layer^51,52^. Testing on surface tension, wettability, droplet size, and photographic image yields better results with **Formula V and Formula VII**. For this reason, **Formula V and Formula VII** were then selected to undergo further tests.

#### Corrosion inhibition effectiveness of formulated metal cutting fluids

**Figure 15:**
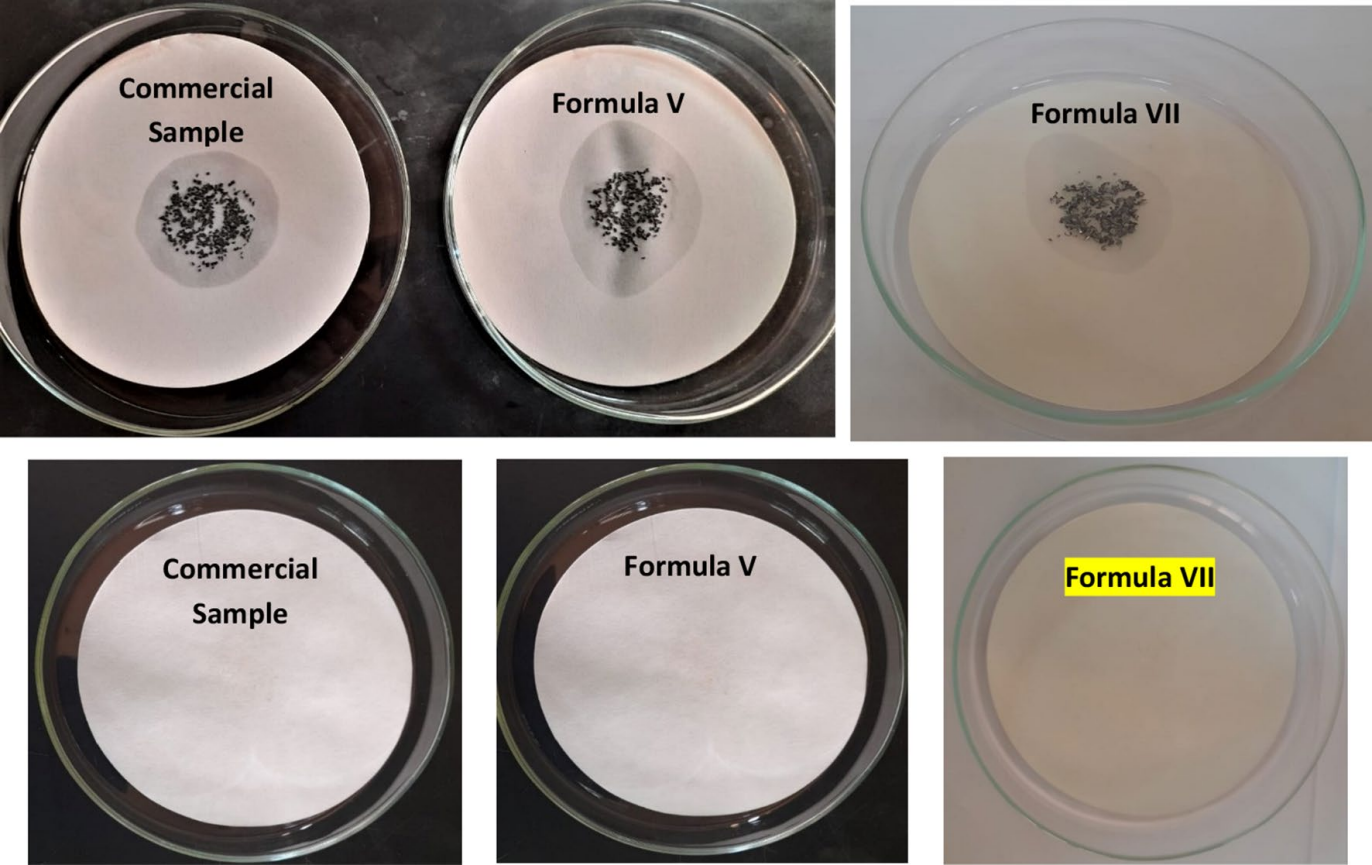
Corrosion test for commercial sample, Formula V and Formula VII after 24 h.

Machine parts and workpieces will be harmed if MCFs used do not prevent corrosion formation. Cutting oil inhibits rust formation but does not cool as effectively as water. The best and least expensive coolant is water, but without rust inhibitors, it rusts parts. Rust inhibitors, which slow down rusting, are now a component of all chemical cutting fluids^46,53^. A new cationic corrosion inhibitor (GCS) has been synthesized in order to investigate the effect of corrosion inhibitors on rust formation during metal-cutting operations. The corrosion test was conducted for the cutting oil/water V/V percentages (5:95). According to the results of the corrosion test, Fig. 15, **Formula V, Formula VII,** and **the commercial sample** demonstrated good protection efficiency. The strong adsorption of the GCS to the metal surface through forming a protective film is likely the reason for protecting the machine parts and tool. The performance of **Formula V** is also significantly influenced by increasing the polyoxyethylene length for the emulsifier GEB, which improves the adsorption on the metal surface.

#### Tribological properties of the formulated metal cutting fluids

Lubricants and cutting fluids are media that help machining operations. They primarily serve to lubricate and cool the area around the cutting zones. Area of contact, stress distribution factor, interfacial temperature, etc. are tribological factors that must be taken into account in a metal-cutting process^4,5^.

The friction coefficient, often known as the coefficient of friction, is a dimensionless quantity whose magnitude represents the relative ease of initiating or sustaining relative motion between two, typically solid, pressing bodies^54^.

**Figure 16:**
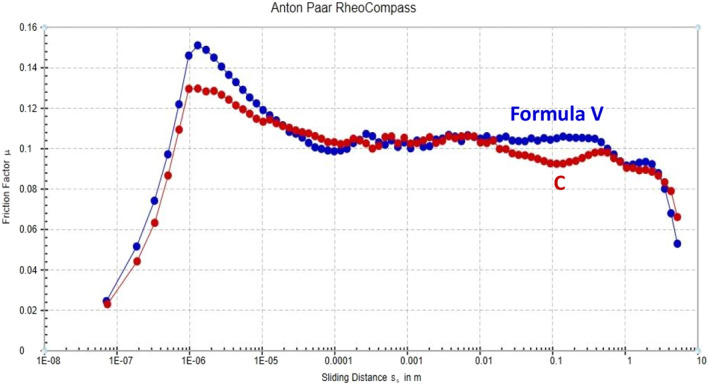
Variation of Friction factor (μ) with Sliding Distance (S) for the Commercial Sample and Formula V under the Applied Load 10 N.

Figure 16 displays the friction coefficient of the test specimens (commercial sample and **Formula V**) calculated from the frictional force of the specimens while sliding against sliding distance for a load of 10 N. It is intriguing to observe that friction typically shows a relatively large peak at short sliding distances. More frictional force may be the cause of this. In contrast, as the sliding distance increases, the friction coefficient decreases as a result of less friction between the ball and the test specimen plates^55,56^.

Based on the data collected, it was determined that **Formula V** gave the workpiece the desired surface quality while reducing friction and limiting tool wear. This observation can be explained by the fluid film boundary that forms between the ball and the three plates. In light of this, the heat generated during sliding and collision stimulates chemical reactions between the nascent surfaces and the MCF, resulting in the formation of a lubricating film^4,5^.

The chemical composition of the surfactants and vegetable oil used had an impact on the adhesion strength of the formed film to the metal substrate as well as the film thickness. At a given load and speed, the film’s thickness and adherence increase, which depresses the coefficient of friction (μ)^57^.

## Conclusion

MCFs were utilized in metalworking machines for a number of purposes, including extending the life of the tools, lowering the thermal deformation of the workpiece, flushing away chips from the cutting zone, and enhancing surface quality. Based on six emulsifiers, six cutting oil formulations were created. The following tests were used to evaluate the cutting oil’s performance: emulsion stability test, corrosion inhibition test, surface tension, contact angle, tribological Properties test, and biodegradability test. The following conclusion is reached as a result of all the evaluated tests:

1. The optimum percentages by volume of cutting oil ingredients are 82% castor oil, 8% emulsifier, 3% corrosion inhibitor, and biocide, 3% coupling agent, and 4% lubricant oil (oiliness).
2. The 15/85, 10/90, and 5/95 oil/water ratios were used to test the stability of the emulsion.
3. Formulas II and V produced stable emulsions at all oil/ water ratios.
4. Economically, the oil/water (5/95) ratio was selected for further investigations.
5. Formula V has good wettability, anti-wear, and surface tension values.
6. The surface active properties, HLB, and synergistic effect of the ingredients all have a significant impact on the performance of the new cutting fluid emulsion.
7. The prepared emulsifier findings were comparable to the benchmark emulsifier data.
8. By contrasting the outcomes of the prepared Formula V with a commercial sample, it was found that the prepared Formula V was comparable to the commercial sample.

## Data Availability

The data used and analyzed during the current study are available from the corresponding author upon reasonable request as long as the request does not compromise intellectual property interests.

## References

[1] Osama M (2017). Recent developments and performance review of metal working fluids. Tribol. Int..

[2] Muralidhar V, Chaganti PK (2020). A review on testing methods of metalworking fluids for environmental health. Mater. Today Proc..

[3] Xue Y (2020). Effects of the chemical structure of surfactants on the stability of naphthenic oil-based metalworking fluids. Chin. Chem. Lett..

[4] Tribology in metal cutting BT - encyclopedia of tribology. in (eds Wang, Q. J. & Chung, Y.-W.) 3837 (Springer US, 2013). 10.1007/978-0-387-92897-5_101458.

[5] Wakabayashi, T. & Inasaki, I. Function of cutting fluids and lubricants BT - encyclopedia of tribology. in (eds Wang, Q. J. & Chung, Y.-W.) 1424–1428 (Springer US, 2013). 10.1007/978-0-387-92897-5_1202.

[6] Kumar CHRV, Ramamoorthy B (2007). Performance of coated tools during hard turning under minimum fluid application. J. Mater. Process. Technol..

[7] Noor El-Din MR, Mishrif MR, Kailas S, Mannekote JK (2018). Studying the lubricity of new eco-friendly cutting oil formulation in metal working fluid. Ind. Lubr. Tribol..

[8] Antonicelli M, Piccininni A, Cusanno A, Lacedra V, Palumbo G (2023). Evaluation of the effectiveness of natural origin metalworking fluids in reducing the environmental impact and the tool wear. J. Clean. Prod..

[9] Nune MMR, Chaganti PK (2019). Development, characterization, and evaluation of novel eco-friendly metal working fluid. Measurement.

[10] Sankaranarayanan R, Krolczyk GM (2021). A comprehensive review on research developments of vegetable-oil based cutting fluids for sustainable machining challenges. J. Manuf. Process..

[11] Byers JP (2017). Metalworking Fluids.

[12] Debnath S, Reddy MM, Yi QS (2014). Environmental friendly cutting fluids and cooling techniques in machining: A review. J. Clean. Prod..

[13] Lodhi APS, Kumar D (2021). Natural ingredients based environmental friendly metalworking fluid with superior lubricity. Colloids Surf. A Physicochem. Eng. Asp..

[14] Singh Lodhi AP, Kumar D, Kaur T, Singh N (2021). Development of lubricious, non-toxic, and corrosion-resistant metalworking fluid: A possible replacement for mineral oil-based fluids. J. Clean. Prod..

[15] Wickramasinghe KC, Sasahara H, Rahim EA, Perera GIP (2020). Green metalworking fluids for sustainable machining applications: A review. J. Clean. Prod..

[16] Liu Z, Zhu G, Dai J, Zhu Y, Lin N (2022). Cellulose nanocrystals as sustainable additives in water-based cutting fluids. Carbohydr. Polym..

[17] Ouellette RJ, Rawn JD (2018). Organic Chemistry: Structure, Mechanism, Synthesis.

[18] Elsharaky EA, El-Tabei AS, El-Tabey AE (2022). The influence of newly synthesized demulsifiers on the interfacial rheological properties of a naturally occurring water/oil emulsion. ACS Omega.

[19] Cremlyn RJ (2002). Chlorosulfonic Acid: A Versatile Reagent.

[20] Moody B (1991). Comparative Inorganic Chemistry.

[21] El-Tabei AS, Hegazy MA, Bedair AH, El Basiony N, Sadeq MA (2021). Experimental and theoretical (DFT&MC) studies for newly synthesized cationic amphiphilic substance based on a naphthol moiety as corrosion inhibitor for carbon steel during the pickling process. J. Mol. Liq..

[22] El-Tabei AS, El-Tabey AE, El Basiony NM (2022). Newly imine-azo dicationic amphiphilic for corrosion and sulfate-reducing bacteria inhibition in petroleum processes: Laboratory and theoretical studies. Appl. Surf. Sci..

[23] Elaraby A (2023). Synthesis of Gemini cationic surfactants based on natural nicotinic acid and evaluation of their inhibition performance at C-steel/1 M HCl interface: Electrochemical and computational investigations. Colloids Surf. A Physicochem. Eng. Asp..

[24] Perez C, Pauli M, Bazerque P (1990). An antibiotic assay by the agar well diffusion method. Acta Biol. Med. Exp..

[25] Jorgensen JH, Turnidge JD (2015). Susceptibility test methods: Dilution and disk diffusion methods. Man. Clin. Microbiol..

[26] Shaban SM, Aiad I, Ismail AR (2016). Surface parameters and biological activity of N-(3-(dimethyl benzyl ammonio) propyl) alkanamide chloride cationic surfactants. J. Surfactants Deterg..

[27] Emulsification by Surfactants. in *Surfactants and Interfacial Phenomena* 336–367 (2012). 10.1002/9781118228920.ch8.

[28] Adetunji CO (2022). Applications of Next Generation Biosurfactants in the Food Sector.

[29] Kruglyakov PM (2000). Hydrophile - Lipophile Balance of Surfactants and Solid Particles: Physicochemical Aspects and Applications.

[30] Watanabe S, Fujita T, Sakamoto M, Kamaru H, Kawahara H (1991). Characteristic properties of cutting fluid additives derived from fatty alcohols. J. Am. Oil Chem. Soc..

[31] Nakagawa H, Watanabe S, Fujita T, Sakamoto M (1998). Characteristic properties of cutting fluid additives made from the derivatives of some polymeric nonionic surface-active agents. J. Surfactants Deterg..

[32] Tomoda H, Sugimoto Y, Tani Y, Watanabe S (1998). Characteristic properties of cutting fluid additives derived from the reaction products of hydroxyl fatty acids with some acid anhydrides. J. Surfactants Deterg..

[33] Heyer P, Läuger J (2009). Correlation between friction and flow of lubricating greases in a new tribometer device. Lubr. Sci..

[34] Gallego R, Cidade T, Sánchez R, Valencia C, Franco JM (2016). Tribological behaviour of novel chemically modified biopolymer-thickened lubricating greases investigated in a steel–steel rotating ball-on-three plates tribology cell. Tribol. Int..

[35] Xu A (2017). Differences in tribological behaviors upon switching fixed and moving materials of tribo-pairs including metal and polymer. Sci. Rep..

[36] Abdel-Hameed HS (2022). Chemical transformation of Jojoba oil and Soybean oil and study of their uses as bio-lubricants. Ind. Crops Prod..

[37] Qin L, Wang XH (2017). Surface adsorption and thermodynamic properties of mixed system of ionic liquid surfactants with cetyltrimethyl ammonium bromide. RSC Adv..

[38] El-Din MRN (2011). Study on the stability of water-in-kerosene nano-emulsions and their dynamic surface properties. Colloids Surf. A Physicochem. Eng. Asp..

[39] Negm NA, Tawfik SM (2014). Characterization, surface properties and biological activity of some synthesized anionic surfactants. J. Ind. Eng. Chem..

[40] Atta AM, Abdel-Rauf ME, Maysour NE, Gafer AK (2010). Water-based oil spill dispersants based on rosin formaldehyde resins. J. Dispers. Sci. Technol..

[41] Rosen MJ, Kunjappu JT (2012). Surfactants and Interfacial Phenomena.

[42] Asadov ZH, Tantawy AH, Zarbaliyeva IA, Rahimov RA (2013). Synthesis of new surface-active ammonium-type complexes based on palmitic acid for removing thin petroleum films from water surface. Egypt. J. Pet..

[43] Israelachvili JN (2011). Intermolecular and Surface Forces.

[44] Boyd J, Parkinson C, Sherman P (1972). Factors affecting emulsion stability, and the HLB concept. J. Colloid Interface Sci..

[45] Al-Sabagh AM (2002). The relevance HLB of surfactants on the stability of asphalt emulsion. Colloids Surf. A Physicochem. Eng. Asp..

[46] Al-Sabagh AM (2012). Investigation of oil and emulsion stability of locally prepared metalworking fluids. Ind. Lubr. Tribol..

[47] Kumari S (2022). Effect of various lubricating strategies on machining of titanium alloys: A state-of-the-art review. Coatings.

[48] Ni J, Feng K, He L, Liu X, Meng Z (2020). Assessment of water-based cutting fluids with green additives in broaching. Friction.

[49] Şirin E, Kıvak T, Yıldırım ÇV (2021). Effects of mono/hybrid nanofluid strategies and surfactants on machining performance in the drilling of Hastelloy X. Tribol. Int..

[50] Tai BL, Dasch JM, Shih AJ (2011). Evaluation and comparison of lubricant properties in minimum quantity lubrication machining. Mach. Sci. Technol..

[51] Hong IK, Kim SI, Lee SB (2018). Effects of HLB value on oil-in-water emulsions: Droplet size, rheological behavior, zeta-potential, and creaming index. J. Ind. Eng. Chem..

[52] Safaya M, Rotliwala YC (2020). Nanoemulsions: A review on low energy formulation methods, characterization, applications and optimization technique. Mater. Today Proc..

[53] El-Din MRN, El-Hamouly SH, Mohamed HM, Mishrif MR, Ragab AM (2013). Formation and stability of water-in-diesel fuel nanoemulsions prepared by high-energy method. J. Dispers. Sci. Technol..

[54] Blau, P. J. Friction coefficient BT - encyclopedia of tribology. in (eds Wang, Q. J. & Chung, Y.-W.) 1304–1306 (Springer US, 2013). 10.1007/978-0-387-92897-5_169.

[55] Gopinath B, Ramesh G (2018). Analysis of sliding distance over friction coefficient and characterization of hybrid aluminium metal matrix composites. Int. J. Emerg. Technol. Innov. Res..

[56] Tuononen A, Kriston A, Persson B (2016). Multiscale physics of rubber-ice friction. J. Chem. Phys..

[57] Pottirayil A, Kailas SV, Biswas SK (2011). Lubricity of an oil in water emulsion in metal cutting: The effect of hydrophilic/lypophilic balance of emulsifiers. Colloids Surf. A Physicochem. Eng. Asp..

